# Facile Preparation of PVDF/CoFe_2_O_4_-ZnO Hybrid Membranes for Water Depollution

**DOI:** 10.3390/polym15234547

**Published:** 2023-11-27

**Authors:** Adriana Popa, Maria Stefan, Sergiu Macavei, Ioana Perhaita, Lucian Barbu Tudoran, Dana Toloman

**Affiliations:** 1 National Institute for Research and Development of Isotopic and Molecular Technologies, 67-103 Donath Street, 400293 Cluj-Napoca, Romania; adriana.popa@itim-cj.ro (A.P.); maria.stefan@itim-cj.ro (M.S.); sergiu.macavei@itim-cj.ro (S.M.); lucian.barbu@itim-cj.ro (L.B.T.); 2 Raluca Ripan Institute for Research in Chemistry, Babes Bolyai University, 30 Fantanele, 400294 Cluj-Napoca, Romania; imperhaita@gmail.com

**Keywords:** CoFe_2_O_4_-ZnO composite nanoparticles, PVDF membrane, magnetic field, photodegradation, Rhodamine B dye

## Abstract

In this investigation, CoFe_2_O_4_-PVDF and CoFe_2_O_4_-ZnO-PVDF hybrid membranes were prepared using a modified phase inversion method in which a magnetic field was applied during the casting process to ensure a uniform distribution of nanomaterials on the membrane surface. Thus, better absorption of light and increased participation of nanoparticles in the photodegradation process is ensured. The influence of nanomaterials on the crystalline structure, surface morphology, and hydrophilicity properties of the PVDF membrane was investigated. The obtained results indicated that the hybrid membrane exhibited significant differences in its intrinsic properties due to the nanomaterials addition. The hydrophilicity properties of the PVDF membrane were improved by the presence of nanoparticles. The photocatalytic decomposition of aqueous Rhodamine B solution in the presence of the prepared membrane and under visible light irradiation was tested. The hybrid membrane containing CoFe_2_O_4_-ZnO on its surface exhibited a high removal rate.

## 1. Introduction

Polymeric membranes are extremely versatile materials being applied in various fields due to their diverse properties induced by the wide variety of structures and properties of polymers. The polymeric membrane possesses high chemical stability, good mechanical strength, flexibility, and porosity, and is easy to prepare involving low-cost fabrication. Therefore, they have been evaluated as separators in domains such as electrochemical energy storage [1,2], CO_2_/gas separation [3], and water treatment [4]. The most prevalently used polymeric membranes are polysulfone (PSF) [5], polyvinylidene fluoride (PVDF) [6,7], and polyvinyl alcohol (PVA) [8].

Research on recycling polluted water using polymeric membranes experienced an important development in recent years. The polymeric membranes represent an effective method to retain the pollutants through filtration (microfiltration, ultrafiltration, nanofiltration), or reverse osmosis [9,10,11]. Despite the many advantages that the membranes possess, the major drawback is the accumulation of pollutant molecules on the membrane surface and pores exacerbated by the hydrophobic character of the surface causing reductions in separation efficiency and, consequently, the membrane lifetime [12]. A facile method to avoid the fouling tendency is the use of membranes modified with nanoparticles. The presence of nanoparticles will change the chemical properties of the membrane surface providing a hydrophilic character [13]. Moreover, using nanoparticles that have photocatalytic properties will give self-cleaning properties to the membranes [14]. Thus, the pollutant molecules from the membrane surface, and also from water, can be degraded through photocatalysis process. Photocatalysis is an advanced oxidation process which takes place under the light irradiation of semiconductor nanoparticles generating reactive oxygen species able to decompose organic pollutant molecules [15]. 

Different approaches have been reported in the literature to obtain hybrid membranes, such as the immobilization of nanoparticles on the membrane surface and nanoparticles blending in the membrane [16]. Membrane surface modification with nanoparticles was successfully achieved using dip-coating; it was reported that PVDF covered with TiO_2_/poly (sodium styrenesufonate) shows an increase in hydrophilicity and photocatalytic activity against Lanasol Blue 3R under UV light [17]. Atomic layer deposition is another technique used to deposit nanoparticles on the membrane surface and pore walls. Thus, uniform TiO_2_/ZnO composite layers were deposited on the PVDF membrane, driving enhanced hydrophilicity, permeability, photocatalysis, and anti-fouling properties [18]. Another strategy is the insitu growth of inorganic photocatalysts on the membrane surface. Binding agents such as polyacrylic acid (PAA), polydopamine (PDA), and its analogues are applied on the membrane facilitating the adhesion and stability of the grown nanoparticles [19]. Thereby, PVDF-P(L-DOPA)-ZnO hybrid membranes were prepared and show efficient degradation of RhB under UV light irradiation [6]. Besides the improvement induced by the presence of nanoparticles, these hybrid membranes present some limitations such as additional pollution caused by the loss of nanoparticles and water flow reduction due to the presence of nanoparticle into the pores [20].

The second approach used to prepare hybrid membrane is the nanoparticles blending into the polymer casting solution before membrane polymerization. This is a very simple and economic method to obtain hybrid membranes. The increase in the photocatalytic activity was obtained by blending different semiconductor nanoparticles such as ZnO [21], MWCNT-ZnO [22], TiO_2_ [23], and ZnS [24] into the polymeric membranes. Yang et al. pointed out the Fe(III)-TiO_2_/polyvinylidene fluoride (PVDF)-blended membrane exhibits a water flux recovery superior to 70% under visible light irradiation [25]. However, the traditional blending method could drive the non-uniform distribution of nanoparticles in the casting solution and the presence of nanoparticles preponderantly inside the membrane. Consequently, the use of an optimized synthesis method is critical. Several recent studies utilized a magnetic field during the casting step of the synthesis to reduce the embedding of the photocatalysts through the migration of nanoparticles to the membrane surface [13,26]. B. Li et al. showed that Fe_3_O_4_-gC_3_N_4_-PVDF have a photocatalytic activity enhanced with 35% compared with a similar membrane prepared with the traditional phase inversion method [27].

Consequently, new membrane compositions which assure boosted photocatalytic efficiency have been attempted. In this work, magnetic CoFe_2_O_4_-ZnO composite nanoparticles are embedded in PVDF polymer to obtain a photocatalytic composite membrane. The membrane was prepared using the phase inversion method assisted by a magnetic field to assure the homogenous distribution of the composite nanoparticles to the membrane surface. CoFe_2_O_4_-ZnO composite nanoparticles were designed to ensure an efficient charge separation during light irradiation. The effect of the composites on the hydrophilicity and photocatalytic activity of the membrane was studied.

## 2. Materials and Methods

### 2.1. Materials

For the preparation of CoFe_2_O_4_-ZnO composite nanoparticles, the following materials and reagents were used: iron nitrate nonahidrate—Fe(NO_3_)3⋅9H_2_O (98% Alfa Aesar), cobalt nitrate hexahydrate—(Co(NO_3_)_2_⋅6H_2_O (98% Alfa Aesar), sodium hydroxide—NaOH (97% Alfa Aesar), absolute ethanol (p.a., Alpha Aesar), zinc acetate (Zn(CH_3_COO)_2_⋅2H_2_O) (Alpha Aesar Thermo Fisher (Kandel) GmbH, Kandel, Germany), diethylene glycol (DEG), and C_4_H_10_O_3_ (Sigma-Aldrich, Merck, KGaA, Darmstadt, Germany). For polymeric membrane preparation, Poly(vinylidene fluoride)-PVDF, Poly(ethylene glycol)-PEG400, N, and N-demethyl formamide–DMF (Sigma-Aldrich, Merck, KGaA, Darmstadt, Germany) were used. Rhodamine B (RhB, Sigma-Aldrich) was used as pollutant. To study the production of reactive oxygen species (ROS), the following reagents were used: 5,5-dimethyl-1-pyrroline N-oxide (DMPO, Sigma-Aldrich), dimethyl sulfoxide (DMSO, VWR International, Radnor, PA, USA) and dimethylformamide (DMF, Sigma-Aldrich). The aqueous solutions were prepared through the use of Milli-Q water through the Direct-Q 3UV system (Millipore, Bedford, MA, USA).

### 2.2. Sample Preparation

#### 2.2.1. Synthesis of CoFe_2_O_4_ and CoFe_2_O_4_-ZnO Nanoparticles 

CoFe_2_O_4_: ZnO (wt% ratios—1:1) composite materials were prepared in two sequential steps starting from preformed cobalt ferrite (CoFe_2_O_4_) nanoparticles and followed by their coverage with ZnO. Magnetic cobalt ferrite nanoparticles were synthesized by the co-precipitation method using Fe(NO_3_)3⋅9H_2_O and cobalt nitrate hexahydrate. The ZnO nanoparticles were grown on CoFe_2_O_4_ nanoparticles by sol-gel process, starting from Zn(CH_3_COO)_2_ × 2H_2_O and DEG:H_2_O (20:1 vol ratio).

#### 2.2.2. Membrane Preparation

Hybrid PVDF membrane was prepared using the phase inversion method. In order to determine the optimal synthesis conditions, the influence of the solvent type and the concentration of the pore-former on the membrane structure were studied. For this purpose, PVDF membranes were prepared using three types of solvents: dimetilformamide (DMF), dimethylacetamide (DMAC), and dimetilsulfoxid (DMSO). The morphological characterization of the obtained membranes is presented in Supplementary Information (Figure S1). Since the use of DMF solvent drove the formation of homogenous pores with an asymmetric structure, we chose this solvent to further study the influence of pore former concentration. The PEG concentration varied between 3% and 8% and the morphological characteristics of the membranes are presented in Supplementary Information (Figure S2). The optimum PEG concentration found was 5%. Consequently, the following composition was chosen for membrane preparation: 15% PVDF powder and 5% polyethylene glycol (PEG-400) were dissolved in N, N-demethylformamide (DMF). 

**Scheme 1:**
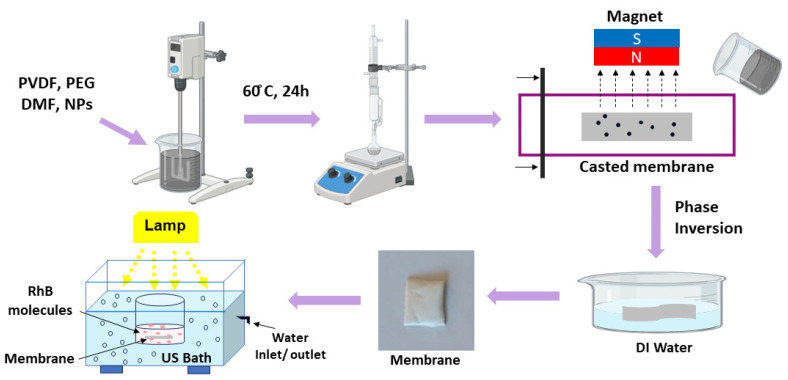
Schematic illustration of membranes preparation and photocatalytic testing setup.

The obtained solution was magnetically stirred at 60 °C for 24 h to ensure the complete dissolution of the polymer. Afterwards, the nanoparticles (0.8%) were added and mechanically stirred until a homogenous solution was obtained. In the next step, the stirring was stopped and the dope was maintained at 60 °C to remove the air bubbles. The degassed dope solution was coated on a substrate using an MSK-AFA-III thin film applicator. Then, a magnetic field (1.4 T) was applied perpendicular to the membrane surface to assure the nanoparticles migration to the surface. The membrane was immersed in the coagulation bath containing DI water to be detached from the substrate. A scheme of the membrane preparation is illustrated in Scheme 1.

### 2.3. Methods

X-ray diffraction (XRD) measurements were performed using a Rigaku-SmartLab automated multipurpose X-ray Diffractometer with Cu-Kα radiation, operating at 45 kV, 200 mA. Fourier transform infrared (FTIR) measurements were performed in Attenuated Total Reflection Mode (ATR) using a JASCO FTIR 4600LE spectrophotometer. The morphology of the composite nanoparticles was evaluated using scanning electron microscopy (SEM). SEM analysis was performed with Hitachi SU-8230 microscope equipped with a cold field emission gun able to accelerate the electron at 30 kV. FMR measurements of powder samples were carried out on a Bruker E-500 ELEXSYS X-band (9.52 GHz) spectrometer at room temperature under identical conditions: microwave frequency of 9.5248 GHz, microwave power 2 mW, modulation frequency of 100 kHz, and modulation amplitude 10 G. The diffuse reflectance spectra were measured using JASCO V570 UV–VIS spectrophotometer, supplied with JASCO ARN-475 absolute reflectivity accessory. The absorbance spectra have been transformed from the recorded reflectance spectra using the spectrometer soft. Specific surface area and porosity measurements were made using a Micromeritics, TriStar II 3020-Surface Area and Porosity Analyzer. Adsorption-desorption isotherms were measured close to the boiling point of nitrogen (77 K).

### 2.4. Photocatalytic Activity Evaluation

The photodegradation of Rhodamine (RhB) synthetic pollutant was carried out in a Laboratory-Visible-Reactor system equipped with a 400 W halogen lamp (Osram) and an ultrasonic bath. The membrane samples (2 × 2 cm^2^) were immersed in 15 mL of aqueous RhB solution (1 × 10^−5^ M). The distance between the lamp and the reaction vessel was fixed at 50 cm. Each degradation experiment was continuously conducted for 5 h. 

To evidence the reactive oxygen species (ROS) generated by the membranes under visible irradiation, EPR, coupled with the spin-trapping probe technique, was employed. DMPO was used as spin-trapping reagent. The membranes (10 mg) were introduced in a solution of DMSO (1 mL) mixed with 0.2 mol/L concentration DMPO and then were subjected to visible irradiation. The samples were prepared immediately before measurements and transferred into the quartz flat cell optimized for liquids measurements. All measurements were performed in the same conditions.

## 3. Results and Discussions 

**Figure 1:**
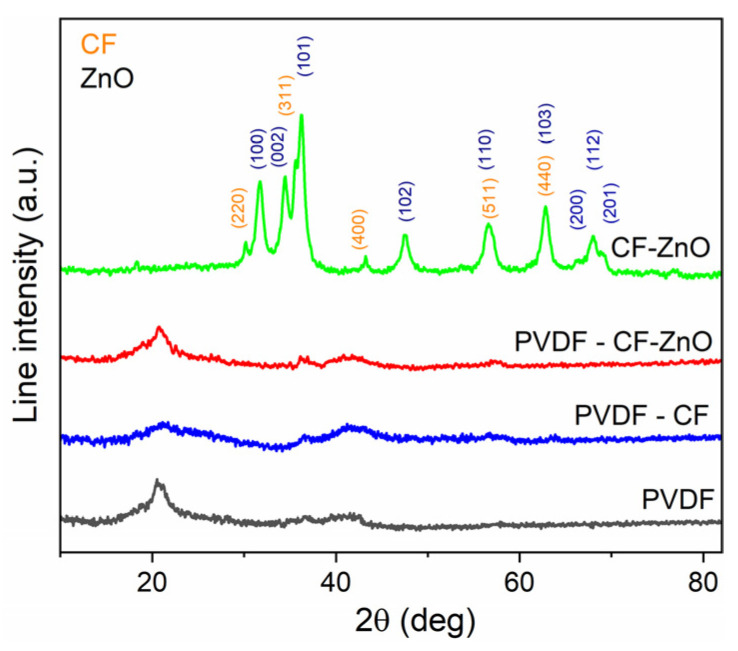
XRD patterns of PVDF, PVDF-CF and PVDF-CF-ZnO membranes.

The crystalline structure of the PVDF before and after nanoparticle blending was analyzed using the XRD technique. PVDF is a semi-crystalline polymer which can crystalize in several polymorphic phases, α, β, γ [28], with mostly a mixture of phases being reported. Figure 1 shows the obtained diffraction patterns. The diffraction pattern of the bare PVDF membrane is characterized by an intense peak at 2θ = 20.7° related with the (110)/(220) reflection of β phase [29]. Beside this, low intensity peaks situated at 18.8° and 41.7°, specific to the (020) and (111) diffraction plans of the α phase, and 36.4° peaks characteristic to the (020) reflection of the β phase, can be identified, which sustain the crystalline nature of the membrane [27].

Nanoparticles addition results in a decrease in line intensity and a line broadening, suggesting that interactions between polymer and nanoparticles exist and influence the PVDF crystalline structure. No diffraction lines specify that CoFe_2_O_4_ or ZnO nanoparticles are observed, probably due to their low concentrations.

**Figure 2:**
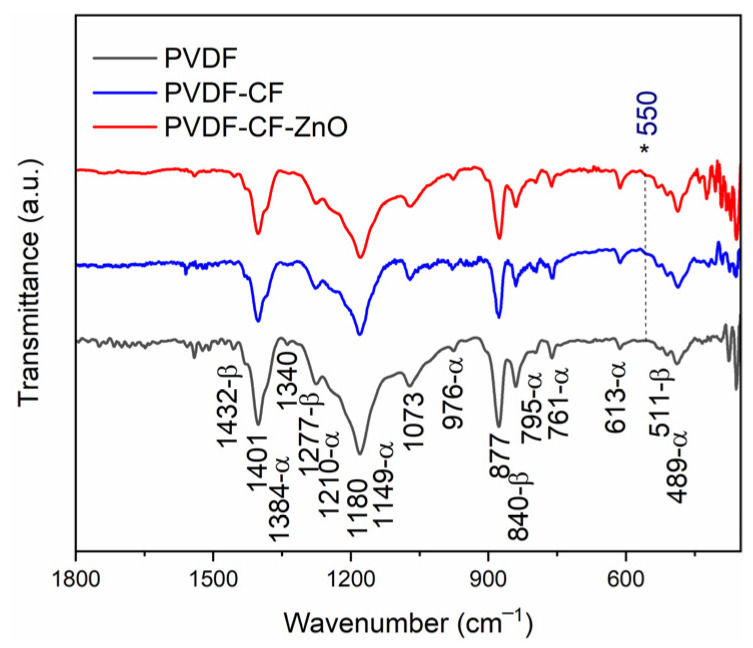
ATRFTIR spectra of the prepared membranes.

For more details regarding the polymorphic phase formed in the PVDF membrane during the synthesis, ATR-FTIR measurements were performed. The obtained spectra of bare and hybrid membranes are shown in Figure 2. 

A visual inspection of the spectra reveals the presence of some similar absorption bands in bare membrane and hybrid membranes. In accordance with XRD results, two polymorph phases are identified in the samples by the presence of a specific band at 761 cm^−1^ and 613 cm^−1^, characteristic to the α phase, and 1277 cm^−1^ specific to β phase. No band shifts after the addition of nanoparticles were observed. The PVDF-CF membrane had a supplementary absorption band of low intensity at 550 cm^−1^ associated with stretching vibrations of the metal-oxygen in the tetrahedral sites from CoFe_2_O_4_ nanoparticles. When CoFe_2_O_4_-ZnO composites are blended in PVDF, a new wide absorption band at 386 cm^−1^ is observed probably due to the Zn–O stretching vibration [30]. In addition, all spectra present strong absorption bands due to the CF_2_ group at 1400 cm^−1^, 1070 cm^−1^, and 876 cm^−1^. Another intense band is observed at 1180 cm^−1^ and is usually attributed to C–F bond stretching vibrations [29]. Besides these, the CH_2_ groups absorption bands of low-intensity can be identified at 1340 cm^−1^, 3016 cm^−1^, and 2978 cm^−1^.

**Figure 3:**
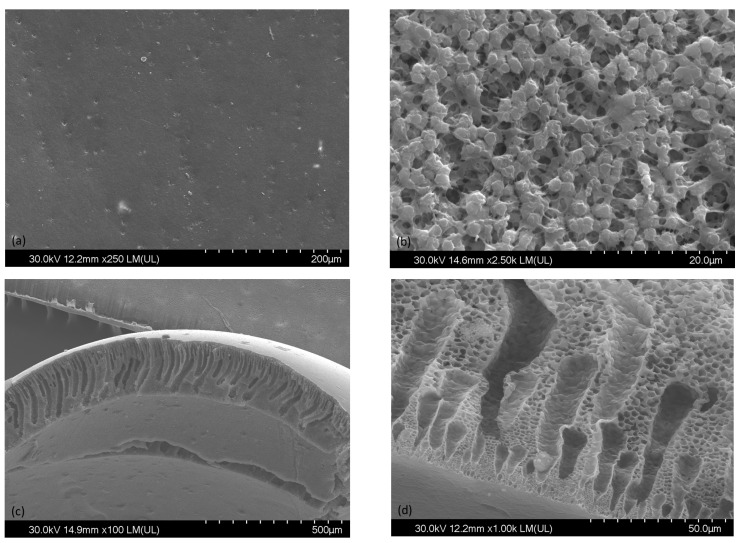
SEM images of bare PVDF membrane (**a**) top side, (**b**) bottom side, (**c**) cross section, and (**d**) enlarged cross section.

The SEM characterization was performed to evaluate the membrane morphology. The obtained images revealing the top side, bottom side, and cross section of the PVDF membrane are illustrated in Figure 3. It can be observed that the membrane has an asymmetric structure divided into three layers. At the top side of the membrane, a dense layer is formed which continues with cavities, having a finger-like structure surrounded by a porous layer. Figure 3c shows a membrane thickness of about 180 μm. The diameters of these cavities vary between 5 and 15 μm. At the bottom side, a porous layer with a globular structure is formed.

**Figure 4:**
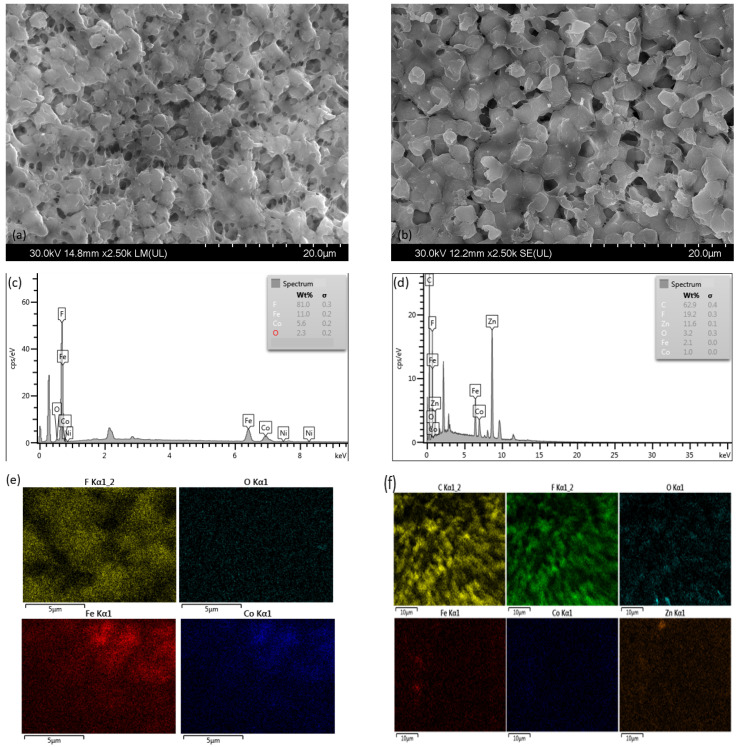
SEM images of bottom layer of (**a**) PVDF-CoFe_2_O_4_ and (**b**) PVDF-CoFe_2_O_4_-ZnO. EDX images and elemental mapping of (**c**,**e**) PVDF-CoFe_2_O_4_ and (**d**,**f**) PVDF-CoFe_2_O_4_-ZnO.

The hybrid membrane morphology is similar to that of bare PVDF. The presence of nanoparticles on the bottom side of the membrane can be observed in Figure 4a,b. The EDX measurements confirm the presence of O, Co, and Fe elements in the PVDF-CoFe_2_O_4_ membrane in addition to C- and F-specific elements of PVDF (Figure 4c). Moreover, Zn, O, Co, and Fe elements were observed in PVDF-CoFe_2_O_4_-ZnO membranes, proving the existence of the composite material on the membrane surface (Figure 4d). The mapping images show an agglomeration of CoFe_2_O_4_ nanoparticles in PVDF-CoFe_2_O_4_ (Figure 4e). In cases of PVDF-CoFe_2_O_4_-ZnO, the composite material is more uniformly distributed on the surface membrane layer (Figure 4f). 

**Figure 5:**
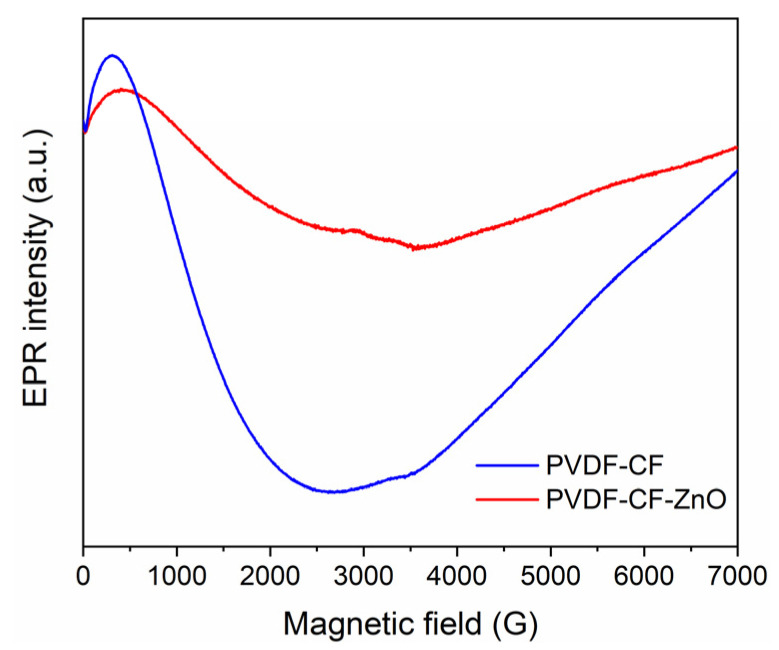
EPR spectra of hybrid membranes.

The presence of cobalt ferrite and cobalt ferrite-zinc oxide nanocomposite in the hybrid membranes can be proved using EPR spectroscopy. The EPR spectra of the hybrid membranes are shown in Figure 5. 

The PVDF-CF membrane shows an intense broad and asymmetric line specific to cobalt ferrite nanoparticles. The line is broad due to the dipolar interaction and to the random orientation of the magnetic nanoparticles having different easy axes and the asymmetry is the result of the random orientation of the magnetic nanoparticles [31]. Superimposed on this signal at a higher magnetic field is a narrow signal, probably due to the Fe^3+^ in the octahedral symmetry sites in the spinel structure of Co-ferrite [32]. The EPR signal of the PVDF-CF-ZnO membrane is similar with that corresponding to the PVDF-CF membrane, but is less intense due to the smaller content of Co-ferrite; additionally, this spectrum shows a signal at g ~ 2 due to the defect states from ZnO [33]

**Figure 6:**
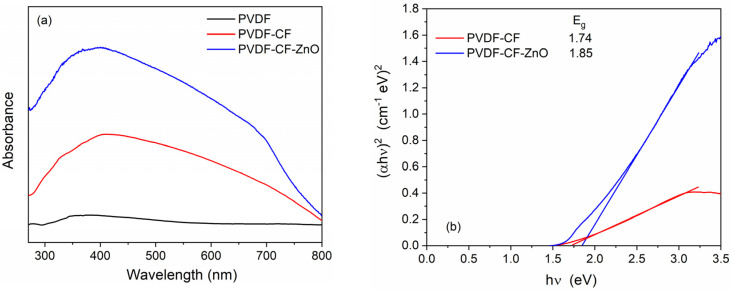
(**a**) UV-Vis absorption spectra of the prepared membranes. (**b**) Tauc’s plots.

Information about the optical properties of the samples was obtained from UV-Vis spectroscopy. The UV-Vis absorption of the spectra are shown in Figure 6a. The PVDF membrane shows low absorbance in the 300–500 nm range. After modification of the PVDF membrane with Co-ferrite nanoparticles and Co-ferrite-ZnO nanocomposite, a broad and intense absorbance appears due to the charge transfer from the O *p* and Co *d* orbitals in strongly hybridized valence band edges into the tetrahedral Fe *d* states at the bottom of the conduction band [34]. 

The band gap energy of the modified membranes was evaluated by the extrapolation of the linear portion of (αhυ)^2^ versus photon energy, hυ, by using the Tauc’s equation (Figure 6b) [35]. The band gap energy of the PVDF-CF membrane is 1.74 eV, a value which was reported previously for CoFe_2_O_4_ nanoparticles [36]. An increase in the band gap energy was obtained for the PVDF-CF-ZnO membrane due to the influence of ZnO, which is known as a wide band gap semiconductor [37]. 

**Figure 7:**
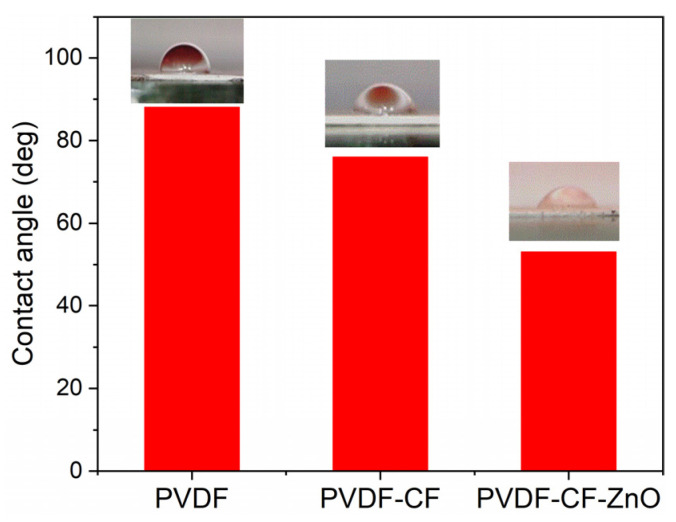
Contact angle for prepared membranes.

An important factor influencing the antifouling properties of a membrane and, implicitly, the filtering capacity is the hydrophilicity. This characteristic can be evaluated by measuring the contact angle between the water and the membrane surface. A small contact angle assures a hydrophilic character and the good antifouling ability of the membrane. In Figure 7 are presented the obtained contact angles for prepared membranes. The bare membrane has a contact angle of 89° but after blending with CoFe_2_O_4_ and CoFe_2_O_4_-ZnO, it decreases to 72 and 50°, respectively. An increase in the hydrophilic character of the membrane through nanoparticles blending can be observed. The best hydrophilicity is exhibited by the PVDF-CF-ZnO membrane, probably due to the hydroxyl groups present on the ZnO nanoparticles surface [38].

Since the properties of our materials can be strongly influenced by the presence of porosity, surface area and porosity measurements were performed. In this respect, the porosity of the membranes was determined using BJH analysis (Barrett, Joyner, and Halenda method) using adsorption/desorption techniques. The general characteristics related to the surface areas (SBET), pore volumes, and pore diameters of PVDF-, PVDF-CF-, and PVDF-CF-ZnO-based samples are presented in Table 1.

**Figure 8:**
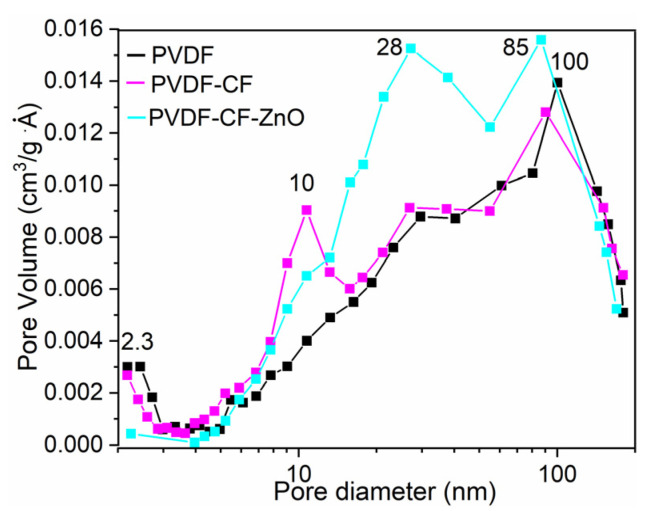
Pore volume distribution curves of PVDF-based samples.

As expected, the specific surface area of the samples increases from 3.27 m^2^/g to 4.01 m^2^/g, together with pore volume from 0.0079 cm^3^/g to 0.0177 cm^3^/g, depending on the nanoparticle type embedded in a polymer matrix. All the samples present a pore distribution in mezzo (2 ÷ 50 nm) and macro-pores (>50 nm) domain, as shown in Figure 8.

The PVDF membrane shows a multi modal pore distribution curve which demonstrates the presence of non-uniform pores, and a predominance of macro-pores around 100 nm. After embedding the CoFe_2_O_4_ and CoFe_2_O_4_-ZnO nanoparticles, the pores become deeper, increase in volume, and are more uniform (Figure 8), as can be seen for the PVDF-CF-ZnO sample. Therefore, based on the evaluated pore size diameter, the membranes have the potential to be used as microfiltration membranes [39].

**Figure 9:**
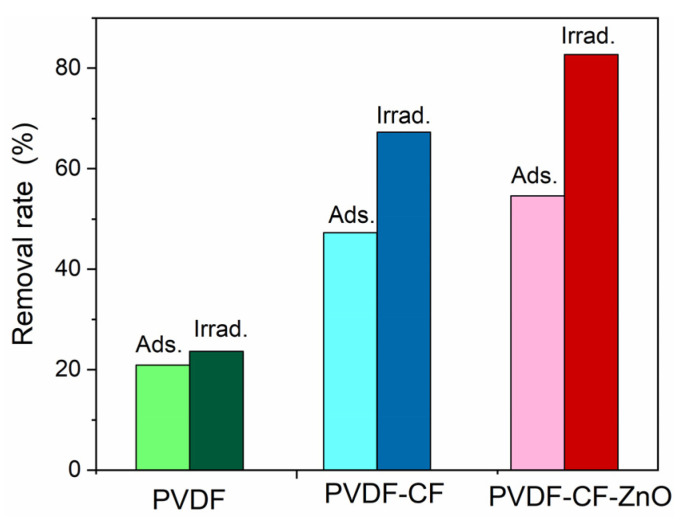
The RhB removal rate of prepared membranes.

The RhB solution removal rate of the prepared membranes was evaluated under visible irradiation. The removal process is a complex one involving adsorption, photolysis, and photocatalysis. Figure 9 shows the removal rate in cases of using all three prepared membranes. The first column shows the adsorption and the second the photocatalysis for every sample after 5 h of dark adsorption and irradiation, respectively.

The difference of about 2.7% between the removal rate in cases of irradiation and dark adsorption for the PVDF membrane represents the photolysis. In the case of hybrid membranes, the removal rate under visible irradiation increases to 67% for PVDF-CF membranes and 83% for PVDF-CF-ZnO. The increases in the adsorption capacity by adding Co-ferrite and Co-ferrite-ZnO nanoparticles is sustained by the results obtained from the contact angle measurements which show the increase in hydrophilicity and by the porosity results which reveal an increase in the surface area. The photocatalytic activity enhancement of the membrane modified with the CF-ZnO nanocomposite can be due to the benefit of heterojunction assuring a delay in the recombination process of the photogenerated charges and also a suitable redox potential [40]. 

**Figure 10:**
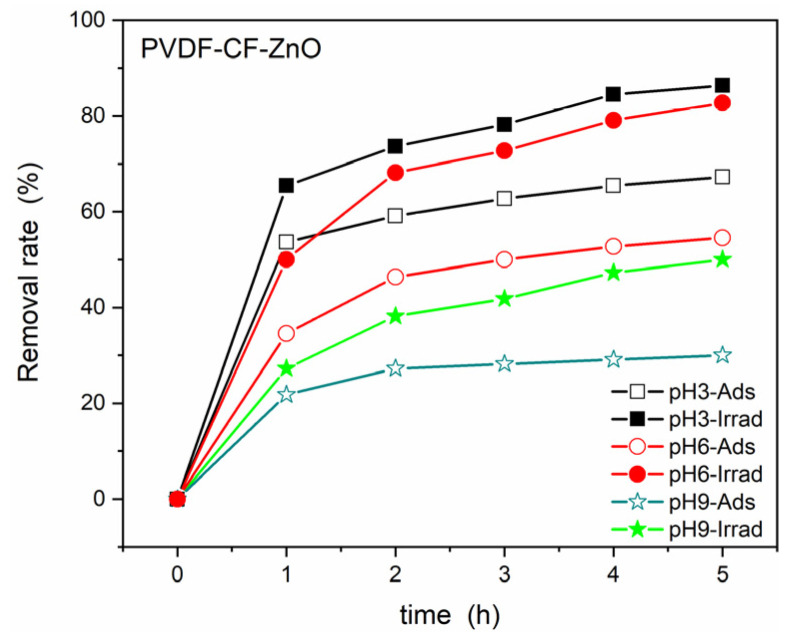
Time evolution of removal rate in function of initial RhB solution pH.

One of the parameters which influence the adsorption and consequently the photocatalytic degradation is the pH of the solution. The pH affects the surface properties of the photocatalyst such as the surface charge and the dissociation of the pollutants [41]. Therefore, the effect of the initial solution pH on the removal rate of the RhB solution was also determined. For the experiments, an initial solution pH in the range 3–9 was used, the membrane surface was (2 × 2 cm^2^), and the obtained results are shown in Figure 10. In a basic solution (pH of about 9) the removal rate is low probably due to the repulsion between the membrane and the pollutant molecules, which are both loaded with a negative charge [42]. This repulsion drives the low adsorption of the pollutant molecules on the catalyst surface, resulting in low photocatalytic activity. The acidic to neutral (pH of 3 and 6) solutions were more effective in degrading than basic solutions. The pH of about 3 provided the highest adsorption capacity and removal rate, but the difference between removal rate and adsorption capacity is lower than in the case of pH 6, meaning that neutral condition is favorable for photocatalytic properties and, therefore, self-cleaning activity.

**Figure 11:**
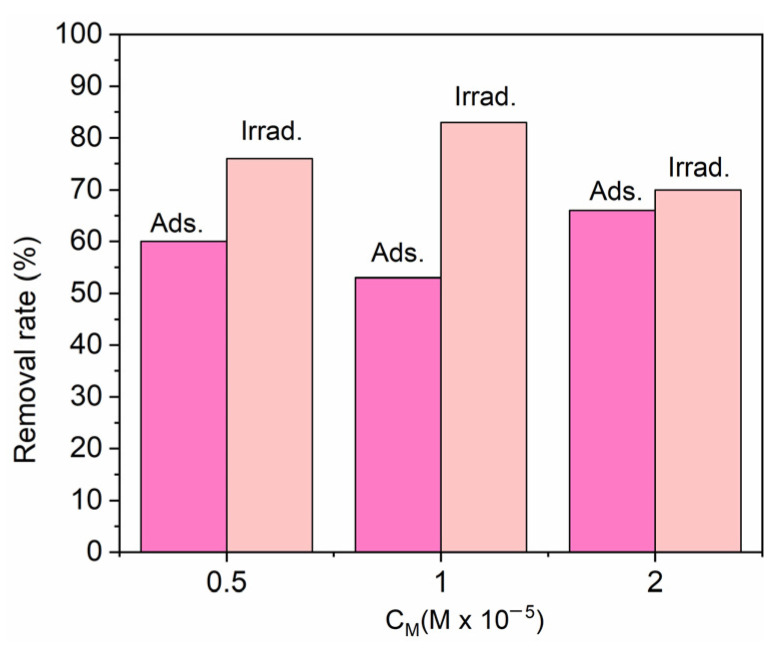
Effects of RhB concentration on the removal rate of PVDF-CF-ZnO sample.

Another parameter that influences the photocatalytic process is the dye dosage. To verify the impact of RhB concentrations on the removal rate of the PVDF-CF-ZnO sample, three dye concentrations of 0.5, 1, and 2 × 10^−5^ M were used. The removal rates obtained after 5 h dark adsorption and visible irradiation in the cases of the mentioned concentrations are shown in Figure 10. In accordance with Figure 11, 1 × 10^−5^ M concentration of RhB is beneficial for photocatalytic activity; this dosage stimulates the free radicals and carriers transfer across the photocatalyst surface. A higher dosage drives an excessive RhB adsorption, blocking the light transmission and consequently hindering the photocatalytic process [43].

**Figure 12:**
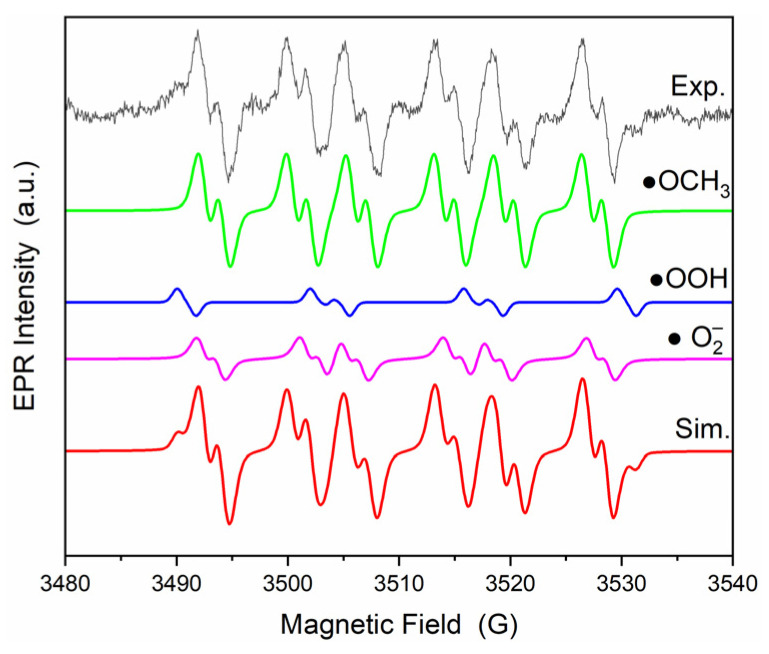
Experimental and simulated spectra of DMPO spin adducts generated by PVDF-CF-ZnO membrane under 25 min visible irradiation.

To give more insights into the photocatalytic mechanism, reactive oxygen species (ROS) generated by the hybrid membranes under visible irradiation were identified using EPR spectroscopy coupled with the spin-trapping method. ROS are able to decompose organic molecules [44]. The experimental spectra of the obtained spin-adducts generated by the hybrid membranes are similar. As an example, the spectrum of the obtained spin adducts generated using the PVDF-CF-ZnO membrane under 25 min visible irradiation is shown in Figure 12. The spectrum is a complex one containing many resonance lines specific to the spin adducts generated under visible irradiation. The simulation of the spectrum was performed to identify the spin adducts which contribute to the spectrum.

The spectra were simulated using the contribution of three components: •DMPO-OCH_3_ (a_N_ = 13.73 G, a_H_ = 11.59 G, a_H_ = 0.99 G, g = 2.0098), •DMPO-OOH (a_N_ = 13.60 G, a_H_ = 11.45 G, a_H_ = 1.06 G, g = 2.0097), and •DMPO-$O_{2}^{-}$ (a_N_ = 13.221 G, a_H_ = 8.04 G, a_H_ = 1.54 G, g = 2.0098) spin adducts. The •DMPO-OCH_3_ spin adduct is due to the interaction between hydroxyl radicals (•OH^−^) and DMSO solvent [45]. •OOH is obtained by the protonation of superoxide radical, $O_{2}^{-},$ resulting from the interaction between the photogenerated electrons and adsorbed O_2_ [46]. Table 2 shows the spin adducts generated by both modified membranes and their relative concentration. Analyzing the relative concentration of the generated spin adducts results in the PVDF-CF membrane generating only superoxide radicals, while the PVDF-CF-ZnO membrane produces, in addition to superoxide, hydroxyl radicals.

Based on the above results, the photocatalytic mechanism can be explained in the following steps: (i) the RhB molecules are attached on the surface and in the pores of membranes; (ii) the irradiation with energy higher than the band gap energy of the Co-ferrite will generate electron–hole pairs; (iii) if generated pairs do not recombine, then, the photo-generated electrons from the conduction band of Co-ferrite will interact with the surface-adsorbed O_2_ resulting •$O_{2}^{-}$ radicals or, in the case of CF-ZnO, they will migrate in the conduction band of ZnO and here they will interact with the adsorbed O_2_ resulting in •$O_{2}^{-}$ radicals. At the same time, a part of the holes from the valence band of ZnO will migrate in the valence band of Co-ferrite, here due to the VB potential of Co-ferrite, which is lower than the oxidation potential of the OH^−^/•OH and H_2_O/•OH redox pair, and these reactions cannot occur [33,47]. Thus, only the remaining holes from VB of ZnO can participate in oxidation reactions. So, this can explain why a PVDF-CF membrane does not generate •OH^−^ radicals.

## 4. Conclusions

In summary, novel PVDF membranes modified with CoFe_2_O_4_ and CoFe_2_O_4_-ZnO nanoparticles were prepared using the phase inversion method combined with the casting process under the action of a magnetic field to ensure a higher number of nanoparticles that are photocatalytic active at the membrane surface. The structural characterization reveals the semicrystalline nature of the hybrid membranes, α and β polymorphic phases being identified. The presence of magnetic nanoparticles was evidenced using EPR spectra which show a broad and asymmetric resonance line specific to ferromagnetic behavior. A broad absorption in visible light was obtained for the hybrid membrane. Moreover, the presence of nanomaterials improves the membrane hydrophilicity. SEM characterization shows the asymmetric structure of the membrane, with no modification being induced by the presence of nanoparticles. All samples present a pore distribution in the mezzo (2 ÷ 50 nm) and macro-pores (>50 nm) domain. The removal rate of RhB was tested in dark and under visible light irradiation. After 5 h of irradiation, the PVDF-CF and PVDF-CF-ZnO showed better removal rate compared with the PVDF membrane. The highest removal rate was being obtained at 83% for the PVDF-CF-ZnO membrane. Two competitive processes are involved in the RhB removal: adsorption and photocatalysis. The photocatalytic activity is due to the addition of CF and CF-ZnO nanoparticles and PVDF-CF-ZnO exhibits the highest efficiency. It was evidenced that in the function of the type of nanoparticles present in the membrane, different reactive oxygen species are formed under light irradiation. PVDF-CF membranes generate especially superoxide radicals, while PVDF-CF-ZnO membranes produce both types of ROS: hydroxyl and superoxide radicals. 

## Figures and Tables

**Table 1:** The textural characteristics for PVDF based samples.

Sample	S_BET_ (m^2^/g)	Pores Volume (cm^3^/g)	D (nm)
**PVDF**	3.27	0.0079	15.63
**PVDF-CF**	4.01	0.0173	24.74
**PVDF-CF-ZnO**	3.94	0.0177	28.56

**Table 2:** The generated spin adducts by PVDF-CF and PVDF-CF-ZnO membranes together with their relative concentration.

Sample	•OCH_3_ (%)	•OOH (%)	$•O_{2}^{-}$ (%)
**PVDF-CF**	0	23	77
**PVDF-CF-ZnO**	30	6	64

## Data Availability

The data presented in this study are available on reasonable request.

## Supplementary Materials

The following supporting information can be downloaded at: https://www.mdpi.com/article/10.3390/polym15234547/s1, Figure S1: SEM image of membrane surface and cross-section prepared using: (a) DMF; (b) DMAC and (c) DMSO solvents. Figure S2: SEM image of membrane surface and cross-section for: (a) 8%PEG; (b) 5%PEG and (c) 3% PEG.
