# Supplementary material for: Facile Preparation of PVDF/CoFe2O4-ZnO Hybrid Membranes for Water Depollution
Source: Polymers (Basel). 2023 Nov 27;15(23):4547. doi: 10.3390/polym15234547 (PMC10708052; doi:10.3390/polym15234547)
Supplement: Supplementary file 1 [file polymers-15-04547-s001.zip › polymers-2732800-supplementary.pdf]

Facile Preparation of PVDF / $CoFe_2O_4$-ZnO Hybrid Membranes for Water Depollution

Adriana Popa, Maria Stefan, Sergiu Macavei, Ioana Perhaita,
Lucian Barbu-Tudoran, Dana Toloman

- *The influence of solvent on the membrane morphology*

In order to determine the optimal synthesis conditions, the influence of the solvent type and the concentration of the pore-former on the membrane structure was studied.

Thus, PVDF membranes were prepared using three types of solvents: dimetilformamide (DMF), dimethylacetamide (DMAC) and dimetilsulfoxid (DMSO). The SEM images of the surface and cross-section of the obtained membranes are shown in Fig. The membrane prepared with DMF solvent presents at the bottoms side a uniform distribution of spherical pores with mean diameter of 2 µm. The use of DMAC solvent drive to the formation of elongated pores with a length of 4 µm. A non-uniform distribution of pores was obtained for the membrane prepared with DMSO solvent. The pores diameter varies between 0.7 µm and 3 µm.

The cross-section analysis of the membranes reveals an asymmetric structure for the sample prepared with DMF and DMAC solvents. In this case, cavities are developed on a considerable thickness of the membrane. In contrast, the DMSO solvent favorized the formation of the porous structure with few and short cavities.

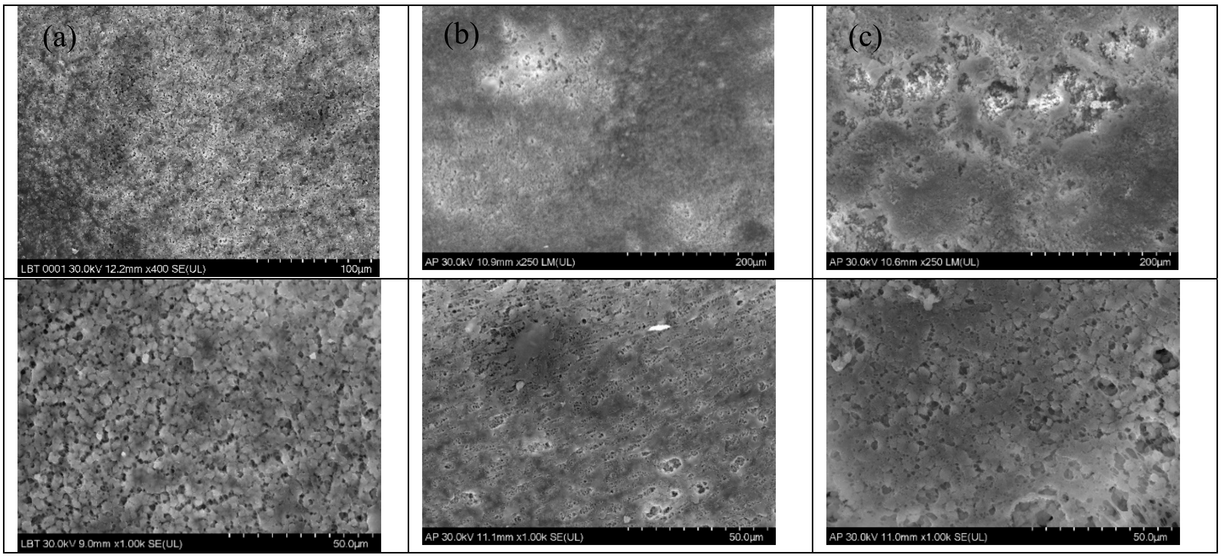

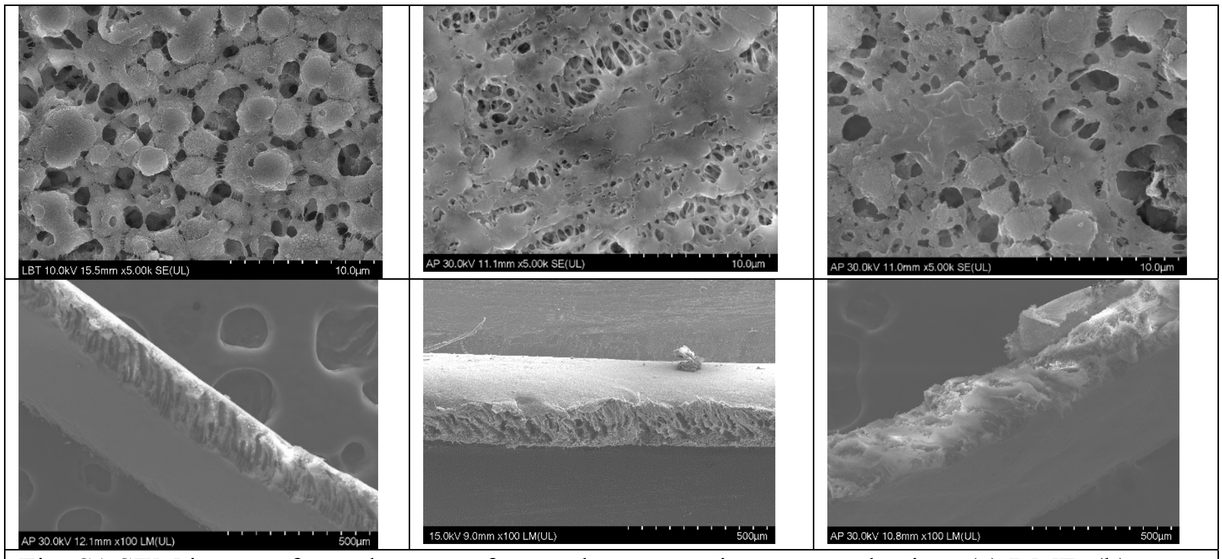

Fig. S1 SEM image of membrane surface and cross-section prepared using: (a) DMF; (b) DMAC and (c) DMSO solvents.

Consequently, the use of DMF solvent drive to the formation of homogenous pores with an asymmetric structure. Moreover, this preparation condition drive to the stabilization of α and β polymorphic phases.

- *The influence of pore former on the membrane morphology*

Taking into account the previously presented results we choose this solvent to study forward the influence of pore former concentration. Various concentrations of PEG were used to prepared PVDF membrane. In Fig S3 are presented the SEM images of the obtained membranes. The use of 8% PEG conduct to the formation of visible pores on the membrane surface and of finger-like structure in all membrane section. In contrast, for 3% PEG a more compact surface was observed with lower pore density. The cross-section image reveals the formation of the asymmetric structure with finger-like and a sponge structure at the surface

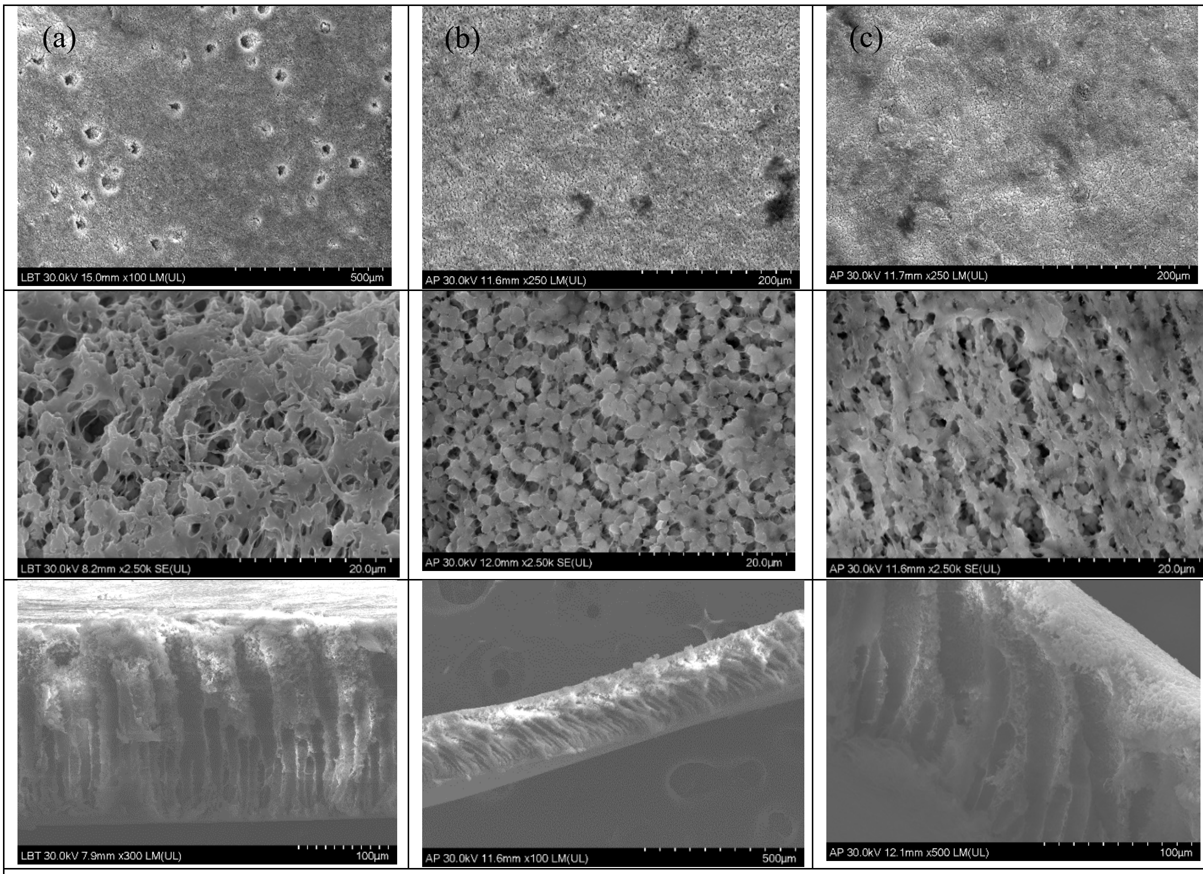

Fig. S2 SEM image of membrane surface and cross-section for: (a) 8%PEG; (b) 5%PEG and (c) 3% PEG
